# 1043. Activity of Mecillinam Against Enterobacterales Isolates Collected From Patients With Urinary Tract Infections (UTIs) in the USA During 2019

**DOI:** 10.1093/ofid/ofab466.1237

**Published:** 2021-12-04

**Authors:** Stephen Hawser, Ian Morrissey, Anne Santerre Henriksen

**Affiliations:** 1 IHMA, Monthey, Valais, Switzerland; 2 IHMA Europe, Monthey, Valais, Switzerland; 3 Maxel Consulting ApS, London, England, United Kingdom

## Abstract

**Background:**

Mecillinam is a β-lactam antibiotic that exerts its antibacterial activity by binding to penicillin-binding protein 2. In the USA, intravenous (IV) mecillinam is in development for the treatment of complicated UTIs in the hospital setting and as step-down therapy transitioning from IV mecillinam to oral pivmecillinam so that patients can continue treatment at home. To support the clinical development of mecillinam in the USA for the treatment of both complicated and uncomplicated UTI, this observational study investigated the activity of mecillinam against Enterobacterales isolates from patients with UTI in the USA, collected during 2019.

**Methods:**

This study evaluated the activity of mecillinam and other antimicrobial agents against 1075 selected Enterobacterales clinical isolates collected from patients with UTI in the USA during 2019. Antibiotic activity (minimum inhibitory concentration [MIC]) was determined by Clinical & Laboratory Standards Institute (CLSI) agar dilution methodology, and susceptibility was interpreted according to CLSI guidelines.

**Results:**

Among the selected 1075 isolates, producers of extended-spectrum beta-lactamase (ESBL) represented 9.6% of *Escherichia coli* and 50% of *Klebsiella pneumoniae*. Ninety-five percent of the isolates tested were susceptible to mecillinam (Table 1). The MIC_50_ and MIC_90_ values for mecillinam were 0.25 and 2 µg/mL, respectively. Fosfomycin MIC_50_ and MIC_90_ were 1 and 32 µg/mL, respectively (97.6% of isolates were susceptible). Mecillinam showed the lowest MIC_90_ value of all single antibiotics tested. The highest MIC_90_ was 128 µg/mL for both nitrofurantoin and cefotaxime. The lowest percentage of resistance was obtained with fosfomycin (1.7%), followed by mecillinam (4%).

Table 1: Summary MIC and susceptibility data for all isolates tested (n=1075)

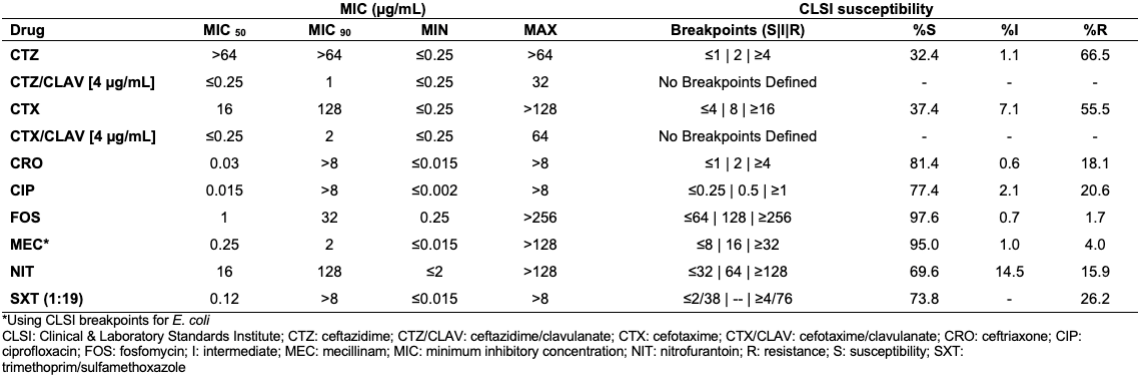

**Conclusion:**

Overall, mecillinam showed excellent activity and a comparable resistance profile to fosfomycin. Resistance rates to ceftazidime, cefotaxime, ciprofloxacin and trimethoprim/sulfamethoxazole of greater than 20% are concerning due to the frequent use of these antibiotics in clinical practice to treat UTIs. Taken together, these data demonstrate that mecillinam has promising activity, with low resistance observed in Enterobacterales species causing UTIs in the USA. Clinical development of mecillinam in the USA is ongoing.

**Disclosures:**

**Stephen Hawser, PhD**, **Utility Therapeutics** (Grant/Research Support) **Ian Morrissey**, **Utility Therapeutics** (Grant/Research Support) **Anne Santerre Henriksen, MS**, **Advanz** (Consultant)**Shionogi BV** (Consultant)**UTILITY Therapeutics** (Consultant)

